# Hypogammaglobulinaemia in patients with ANCA-associated vasculitis treated with rituximab

**DOI:** 10.3389/fmed.2026.1852606

**Published:** 2026-06-16

**Authors:** Jens Rathmann, Hiba Kadhem, Mårten Segelmark, David Jayne, Aladdin J. Mohammad

**Affiliations:** 1 Department of Clinical Sciences Lund, Rheumatology, Lund University, Lund, Sweden; 2 Department of Clinical Sciences Lund, Nephrology, Lund University, Lund, Sweden; 3 Department of Medicine, University of Cambridge, Cambridge, United Kingdom; 4 Department of Clinical Sciences Lund, Clinical Epidemiology Unit, Lund University, Lund, Sweden

**Keywords:** ANCA-associated vasculitis, granulomatosis and polyangiitis, hypogammaglobulinaemia, rituximab, severe infection

## Abstract

**Objective:**

This study aimed to characterize the occurrence of hypogammaglobulinaemia (HG) in rituximab (RTX)-treated patients with ANCA-associated vasculitis (AAV) in a population-based Swedish cohort.

**Methods:**

Patients with incident AAV (1997–2019) in southern Sweden who received RTX for induction or maintenance therapy were included in this study. Medical records were reviewed to assess the incidence of HG (defined as total IgG < 6.7 g/L; mild 5–6.7, moderate 3–4.9, severe <3), as well as demographics, serology, and treatment history. Incidence rates and predictors of HG were analyzed. Follow-up was extended from the first RTX administration to the onset of HG, death, or the end of the study period (March 2023).

**Results:**

Eighty-four patients [51% female; granulomatosis with polyangiitis (GPA), *n* = 57; microscopic polyangiitis (MPA), *n* = 25; eosinophilic granulomatosis with polyangiitis (EGPA), *n* = 2] received RTX and were followed for a total of 236 person-years (py). HG occurred in 38 patients (45%), yielding an incidence rate of 16.1 (95%CI 11–21.2) per 100 py. A sensitivity analysis excluding 14 patients with preexisting HG identified 24 incident HG cases, corresponding to an incidence rate of 17.6 (95%CI 10.6–24.7) per 100 py. Among those who developed HG, the condition was mild in 26 (68%) patients, moderate in 10 (26%), and severe in two patients (5%). Baseline IgG levels were significantly lower in patients who subsequently developed HG, whereas age, clinical characteristics, and other laboratory findings did not differ between groups. Severe infections occurred in 23 patients (35%); five of these infections occurred after the onset of HG, and three occurred before HG onset. The median time to HG development was 3.5 (IQR 0.7–6) months. A lower baseline IgG level was identified as an independent predictor of HG (HR 0.85 per 1 g/L increase; 95%CI 0.76–0.95).

**Conclusion:**

HG is a common complication in RTX-treated patients with AAV; however, in this cohort, it was not associated with an increased risk of severe infections. A low baseline IgG level was an independent predictor of subsequent HG development, underscoring the importance of close IgG monitoring both before and during RTX therapy.

## Introduction

Antineutrophil cytoplasmic antibody (ANCA)-associated vasculitides (AAV) are a group of inflammatory, multisystem diseases affecting small and medium-sized blood vessels. Three subtypes—granulomatosis with polyangiitis (GPA), microscopic polyangiitis (MPA), and eosinophilic granulomatosis with polyangiitis (EGPA)—are defined according to phenotypical, serological, and histopathological criteria (1). AAV are rare diseases with a combined annual incidence rate of 30 cases per million adults and a prevalence of 428 cases per million (2). These diseases were often fatal (3) before the introduction of immunosuppression consisting of glucocorticoids (GC) (4) and cytotoxic drugs (5–7). Although these therapies considerably improved prognosis, patients became prone to treatment-induced toxicities, such as infection, cancer, diabetes, and osteoporosis. In recent decades, treatment has undergone considerable changes through large international randomized clinical trials, yielding standardized treatment protocols, reducing treatment toxicities (8), and introducing new treatment options with modern biologic immunomodulating drugs, such as rituximab (RTX) (9, 10). The latter is a B-cell depleting agent that does not exhibit the same toxicity profile as earlier standard treatment with cyclophosphamide. Studies have demonstrated a comparable rate of infections (9, 10). A potential reason for this is the occurrence of hypogammaglobulinaemia (HG), which has been shown to be associated with infections (11–13). HG commonly occurs in RTX-treated patients (13, 14). Prior studies in patients with AAV have identified older age, glucocorticoid use, prior cyclophosphamide exposure, and female sex as factors associated with HG (15). HG can be transient or persistent, and its frequency varies across different autoimmune diseases (16). HG cannot be attributed to B-cell depletion alone; disease-specific mechanisms appear to contribute, as an increased risk is observed in AAV but not in rheumatoid arthritis (RA) or connective tissue disease after RTX treatment (17). The reported frequency of HG after RTX for the treatment of autoimmune disease varies, with rates between 16 and 56% (13, 18–20). It occurs more frequently after RTX treatment in AAV than in other autoimmune diseases (14, 16). A systematic literature review addressing the role of immunoglobulin replacement therapy (IGRT) concluded that there is currently no agreement on a specific IgG threshold for initiating IGRT, and that a clear distinction between purely numerical HG and symptomatic (infection-associated) HG is lacking. The decision to start IGRT in patients with autoimmune disease should be based on individual infection risk, a history of recurrent bacterial infections, and low IgG levels (21). In this study, we aim to characterize the occurrence of HG, identify predictors, and explore its association with severe infections in a population-based cohort of patients with AAV from Southern Sweden.

## Methods

### Study design, population, and area

In this retrospective cohort study, adult cases were identified from a population-based cohort comprising 374 patients with incident AAV diagnosed between 1997 and 2019 in a defined geographical area in Southern Sweden. Patients had a clinical diagnosis of AAV and were classified into one of the three AAV subtypes using the European Medicines Agency (EMA) algorithm (22) as described previously (2). Patients who had received RTX either as induction or maintenance therapy were identified via digital records. All patients were followed from the date of their first RTX infusion to the date of HG onset, death, or the end of study (March 2023). If HG was already present at baseline, the latter two apply.

### Data collection

The AAV cohort has been studied in several studies (2, 23); therefore, baseline demographics and clinical and laboratory data from the time of AAV diagnosis were collected before the start of this study. In addition, a data collection sheet specifically designed for this study was used to focus on RTX treatment, including dosing and frequency, and on immunoglobulin levels, whenever available.

Data collected specifically for this study included dates and doses of RTX, including cumulative exposure, as well as laboratory data at RTX initiation and during follow-up. Laboratory data comprised full blood count, inflammatory parameters, immunoglobulin levels, ANCA-serology, creatinine, and GFR were also collected at RTX initiation. In addition, data on concomitant medications, such as GC doses or treatment with other maintenance agents, were collated.

### Definitions

HG was defined as an IgG level below 6.7 g/L and was further divided into mild (IgG range 5–6.7 g/L), moderate (IgG range 3–4.9 g/L), and severe HG (IgG below 3 g/L). For the other immunoglobulins, low values were defined according to the normal range given by our local laboratory, i.e., IgA (< 0.88 g/L, normal range 0.88–4.5 g/L) and IgM (<0.27 g/L, normal range 0.27–2.1 g/L). Severe infection was defined as an infection event requiring hospitalization and intravenous antimicrobial treatment. End-stage kidney disease (ESKD) was defined as ongoing dialysis or successful renal transplant.

#### Treatment protocols for AAV

We follow our regional guidelines as well as international recommendations (EULAR) concerning the frequency and dosing of RTX; the same applies to GC doses and tapering recommendations. A dose of 1 g RTX is given intravenously on days 1 and 14 (slightly longer intervals can apply due to logistical problems, 2 doses of RTX 1 g within a month were considered induction treatment, as shown in Table 1 for induction treatment, and then a dose of 500 mg is given every 6 months as maintenance treatment). All patients received infection prophylaxis with trimethoprim-sulfamethoxazole (TPM–SMX) 80/400 mg or recommended alternatives. IGRT was used in only a few cases with severe HG and recurrent infections. Data on IGRT at an individual level could not be obtained and is not included in this report.

**Table 1:** Demographics, serology, and clinical parameters in 84 patients with AAV treated with Rituximab (RTX).

Variables	All *n* = 84	HG *n* = 38	No HG *n* = 46	*p*
Age at AAV diagnosis, yrs., mean ±SD	59.32 ± 16	59.42 ± 14	59.24 ± 17	0.9
Sex, Female, *n* (%)	43 (51.2)	19 (50)	24 (52.2)	1.0
Age at first RTX treatment, yrs., mean ±SD	61.8 ± 15	62.2 ± 13	61.5 ± 17	0.8
Diagnosis, GPA/MPA/EGPA	57/25/2	28/9/1	29/16/1	
PR3–ANCA +, *n* (%)	58 (69.0)	27 (71.1)	31 (67.4)	0.7
MPO–ANCA +, *n* (%)	24 (28.6)	10 (26.3)	14 (30.4)	0.7
RTX induction, *n* (%)	70 (83.3)	35 (92)	35 (76)	0.08
Plasma exchange, *n* (%)	14 (16.7)	5 (13.2)	9 (19.6)	0.5
Cyclophosphamide, *n* (%)	65 (77.4)	30 (78.9)	35 (76.1)	0.7
BVAS at diagnosis, median (IQR)	16.5 (12–19)	16 (12–22.5)	17 (12–19)	0.36
Haemoglobin g/L mean ±SD	120 ± 17.9	110 ± 21.2	112 ± 18.1	0.8
White blood cell count mean ±SD	11.3 ± 3.94	13.3 ± 5.5	12.33 ± 4.3	0.36
Platelet count mean ±SD	325.5 ± 120.3	434.8 ± 164.5	382 ± 149.0	0.13
CRP, mg/L, median (IQR)	5.7	5	6.3	0.5
ESR, mm/h mean ±SD	39.7 ± 28.3	60 ± 30	60.5 ± 26.7	0.9
S–creatinine, μmol/L, median (IQR)	97 (74–153)	100 (82.5–144)	88.5 (72.7–176.2)	0.21
eGFR mL/min x1,73m^2^, median (IQR)	62 (38–76)	58 (34–74)	70 (43.5–77)	0.27
End-stage kidney disease, *n*	3	1	2	1.0
Organ involvement at AAV diagnosis, *n* (%)				
General	71 (84.5)	34 (89.5)	37 (80.4)	0.3
Ear-nose-throat	46 (54.8)	24 (63.2)	22 (47.8)	0.2
Cutaneous	10 (11.9)	3 (7.9)	7 (15.2)	0.3
Mucous membranes	10 (11.9)	6 (15.8)	4 (8.7)	0.5
Chest	54 (64.3)	22 (57.9)	32 (69.6)	0.3
Cardiovascular	5 (6.0)	4 (10.5)	1 (2.2)	0.2
Renal	56 (66.7)	26 (68.4)	30(65.2)	0.6
Nervous	16 (19.0)	10 (26.3)	6 (13.0)	0.1
At time of first RTX treatment				
Prednisolone, mg/day	30.3 ± 21.3	35.5 ± 21.9	28.4 ± 20.8	0.3
IgG g/L mean±SD	9.4 ± 3.5	8.5 ± 3.4	10.2 ± 3.4	0.03
IgA g/L mean±SD	2.3 ± 1.2	1.9 ± 0.8	2.5 ± 1.3	0.02
IgM g/L mean±SD	0.92 ± 0.7	0.8 ± 0.7	1.0 ± 0.07	0.06

GPA, granulomatosis with polyangiitis; MPA, microscopic polyangiitis; EGPA, eosinophilic GPA; eGFR, estimated glomerular filtration rate (using MDRD); BVAS, Birmingham vasculitis activity score (0–63), PR3: proteinase 3, MPA: myeloperoxidase. RTX: rituximab, IgG/A/M: immunoglobulin G/A/M. Data on immunoglobulins prior to RTX available were in 80 cases; 14 (17%) cases show Hypogammaglobulinaemia before RTX (12 mild, 1 moderate, 1 severe), and 14 continue to exhibit HG after RTX.

### Statistical analysis

Descriptive data are presented as frequency and percentage. Normally distributed data are presented as mean and standard deviation (SD), and non-normally distributed data are presented as median with interquartile range (IQR). Categorical data were analyzed with chi-square or Fisher’s exact test as appropriate. Continuous variables were compared between groups with and without HG using Student’s *t*-test for normally distributed data or the Mann–Whitney U test for non-normally distributed data. A *p*-value of < 0.05 was considered significant.

For the analysis of predictors of HG, the hazard ratio (HR) for the occurrence of HG was estimated using Cox regression analysis. Potential predictors were selected based on clinical relevance or findings from earlier studies. The following variables were studied in a univariable and multivariable model: age at AAV diagnosis, sex, renal involvement at diagnosis, serum creatinine at diagnosis, as well as GC dose and IgG level at the time prior to the first RTX treatment.

For the estimation of the incidence rate of HG, the number of HG events was divided by the total person-years (PY) of follow-up. The incidence rate of HG was calculated for all cases with HG after RTX initiation and was repeated after excluding cases with already existing HG at RTX initiation (sensitivity analysis). Follow-up time is presented in PY, summing up the follow-up time of all patients from the date of the 1st RTX to either HG-event, death, or the end of study (March 2023). All statistical analyses were conducted using SPSS version 28 for Windows.

### Ethics

The study was conducted in accordance with the Declaration of Helsinki and was approved by the Regional ethical review board in Lund, Sweden (2010/517).

## Results

Of 374 patients, 84 (51% female) received RTX as either induction and/or maintenance treatment. The median disease duration at the time of RTX treatment was 7.6 months (IQR 1.3–19.5), and most cases were treated for relapsing or refractory disease. The mean age at diagnosis of AAV was 60 years (SD ± 15.6). Fifty-eight patients were PR3-ANCA positive, 24 were MPO–ANCA positive, and two were ANCA-negative. Patients were classified into GPA = 57, MPA = 25, and EGPA = 2. RTX was used as an induction agent in 70 patients (81%); 60 patients (71%) were not treatment-naïve to other immunosuppressants, mainly cyclophosphamide. These patients had previously received either a full intravenous induction cycle or oral cyclophosphamide at any time before RTX, or they had failed CYC treatment and received RTX as an alternative near diagnosis. Prior immunosuppressive therapies included methotrexate as an induction agent in four patients, cyclophosphamide in 60 patients, and plasma exchange in 14 patients. The median time of follow-up from RTX to HG onset, death, or the end of the study was 14 months (IQR 2.3–63.8). Altogether, 623 infusions of RTX were given, with a mean number of treatments per patient of 6 courses (range 1–24).

### Hypogammaglobulinaemia

At least one episode of HG at any time after RTX was observed in 38 patients (45%; 50% female) during 236 years of follow-up. Twenty-eight patients had GPA, nine had MPA, and one had EGPA. There were two (5%) cases with severe HG, 10 (26%) with moderate, and 26 (68%) with mild HG. The mean age at the time of RTX initiation in patients with later HG development was 62.2 years (SD ± 13) compared to 61.5 years (SD ± 17) in patients without later HG. The median time from RTX initiation to onset of HG was 3.5 months (IQR 0.7–6). At RTX initiation, the mean IgG level was 9.4 (±3.5) g/L, IgA was 2.3(±1.2) g/L, and IgM was 0.92 (±0.7) g/L for all cases. HG cases exhibited lower immunoglobulins at the time of RTX initiation than non-HG cases (Table 1). Most patients who developed HG did so during the first year after RTX initiation (*n* = 23; 60% of all patients with HG). There were no differences in terms of disease phenotype, ANCA-serology, disease activity, or renal function at the time of diagnosis of AAV between cases who subsequently developed HG and those who did not. A lower baseline IgG level at the initiation of RTX treatment was observed in patients who had previously received cyclophosphamide, with a mean IgG of 8.7 g/L vs. 11.3 g/L. Cases that received RTX as induction treatment were significantly more likely to develop HG during follow-up (*p* = 0.04). No other differences in treatment were observed between the two groups (Table 1).

### Incidence rate and predictors of HG

The incidence rate for HG was 16.1 (95% CI 11–21.2) per 100 person-years of follow-up. The incidence rate was similar in men and women; 16.8/100 person-years (95% CI 9.3–24.4) vs. 15.4/100 person-years (95% CI 8.5–22.4), and was similar among patients with PR3 and MPO-positive disease.

A higher baseline IgG level at RTX initiation was associated with a reduced risk of HG, with each 1 g/L increase in IgG conferring a 15% lower hazard for HG (HR 0.85; 95%CI: 0.77–0.95). This remained the only independent predictor in the multivariable analysis (Table 2), while prior cyclophosphamide exposure was not significantly associated with HG. Fourteen patients exhibited HG prior to RTX initiation (12 mild, 1 moderate, 1 severe), and all 14 continued to exhibit HG after RTX. When excluding these patients from the incidence calculation and only including the remaining 24 patients with new-onset HG after RTX, the incidence rate remained statistically unchanged [17.6 per 100 py (95% CI 10.6–24.7)].

**Table 2:** Predictors of Hypogammaglobulinaemia in 84 patients with AAV treated with rituximab (RTX).

Predictors	Univariable analysis		Multivariable analysis	
	HR (95% CI)	*p*	HR (95% CI)	*p*
Age at first RTX (10-year increments)	1.07 (0.86–1.33)	0.54	0.95 (0.73–1.23)	0.7
Sex (male)	1.07 (0.6–2.03)	0.8	0.99 (0.51–1.93)	1.0
Renal involvement at AAV diagnosis	1.34 (0.68–2.66)	0.4	1.18 (0.54–2.58)	0.7
S-creatinine at diagnosis (100 μmol/L increments)	1.12 (0.85–1.47)	0.4	1.1 (0.83–1.46)	0.5
Prednisolone dose at first RTX (10 mg increments)	1.05 (0.9–1.2)	0.5	1.05 (0.89–1.24)	0.5
IgG at start of first RTX (1 g/L increments)	0.85 (0.77–0.95)	0.004	0.84 (0.74–0.95)	0.004

AAV, ANCA-associated vasculitis; HR, Hazard ratio; CI, Confidence interval.

### Severe infections

Twenty-three (27.4%) of the 84 patients who received RTX experienced at least one severe infection during follow-up. Among these 23 patients who experienced severe infections, 15 (65%) belonged to the non-HG group, and eight (35%) belonged to the HG group (*p* = 0.3). Pneumonia was the most common infection, accounting for nearly 40% of cases. The median time from RTX initiation to the first severe infection was 5.4 months (IQR 1.5–24.2), with no significant difference between the groups.

### Renal outcome

Among patients who developed HG, renal involvement due to AAV was present in 26 cases (68%), compared with 30 cases (65%) among patients without HG. HG was more frequently observed in patients with renal involvement during the first months after RTX treatment. During follow-up, three patients developed ESKD after RTX treatment: one with HG and two without HG.

## Discussion

In this population-based study of patients with AAV treated with RTX, we observed HG in nearly half of the patients, with the highest frequency occurring in the first year after RTX treatment. The majority of cases exhibited mild or moderate HG, and only 5% developed severe HG. In this study, severe infections were not a common problem, as there were no statistically significant differences between patients with HG and those without HG. We also found that a lower baseline IgG level was the only independent predictor of the later occurrence of HG in this cohort.

In this cohort, HG was common, with approximately 40% of cases exhibiting HG within the first year after the initiation of RTX therapy. This early onset highlights the vulnerability period following B-cell depletion and underscores the importance of close and systematic follow-up, including routine monitoring of immunoglobulin levels, in all patients receiving RTX. Although HG has been described previously in RTX-treated AAV and other autoimmune diseases, the timing of onset has been less well characterized, making our findings particularly relevant for clinical practice. In our hospital, immunoglobulin levels are measured prior to every RTX infusion, even in patients with previously normal IgG levels, reflecting a proactive monitoring strategy that may facilitate the early identification of patients at risk. We also observed an incidence rate of 16.1 cases per 100 person-years; to our knowledge, comparable population-based incidence estimates are lacking in the existing literature, limiting direct comparisons but emphasizing the novelty of our data.

HG has been reported in other studies, with frequencies ranging between 16 and 56%, which are broadly comparable to our findings (13, 18–20, 24). However, Tieu et al. reported a substantially higher frequency of HG, including more severe cases (14), which was likely attributable, according to the authors, to a longer follow-up period (mean 8 years) compared to earlier studies; in addition, only patients with IgG < 7 g/L on two occasions were included in the study. These differences underscore the importance of ongoing monitoring for HG in RTX-treated patients, as the risk may accumulate over time and severe cases may only become apparent during extended follow-up.

Several studies have investigated predictors of HG in patients treated with RTX. Among these, low baseline IgG has consistently been associated with HG (25–27). Cortazar et al. (26) reported that baseline IgG at entry into maintenance therapy was the only factor significantly associated with HG. Consistent with these findings, we identified low IgG at RTX initiation as the only independent predictor of HG. We did not observe an effect of glucocorticoid dose at induction or patient age on the development of HG, as reported by Podesta et al. (13). This discrepancy may reflect insufficient data on exact and cumulative glucocorticoid doses in our study. These results highlight the importance of assessing IgG levels prior to RTX therapy, as patients with lower baseline IgG may benefit from closer monitoring and individualized management strategies to mitigate the risk of HG and related complications.

Padoan et al. (20) reported that previous cyclophosphamide (CYC) exposure and a daily prednisolone dose >15 mg were associated with the subsequent development of HG in patients treated with RTX. Similar findings have been reported by other authors. Marco et al. (15) reported that higher CYC exposure and higher cumulative GC exposure were associated with HG development. CYC treatment resulted in a decrease of all immunoglobulin classes, and this was further aggravated by subsequent RTX administration in a study of AAV patients (28). The impact of CYC is thought to reflect a synergistic effect of CYC and glucocorticoids on serum IgG concentrations. However, this was not observed in our study. Our cohort primarily consisted of patients with AAV who had received CYC prior to RTX, as RTX was introduced more routinely for AAV in our region around 2011 and was generally used as a second-line treatment in cases of CYC failure or relapse occurring years after initial diagnosis. Although we observed lower mean IgG levels at the start of RTX in patients with prior CYC exposure, CYC was not an independent predictor of post-RTX HG in our regression analysis. These findings suggest that, while prior CYC may modestly lower IgG levels, baseline IgG remains the key factor in predicting HG, and monitoring strategies should focus primarily on IgG assessment rather than on prior CYC exposure alone.

In the study by Venhoff et al. (28), the influence of CYC on immunoglobulin levels was modest and often recovered after therapy, whereas subsequent or simultaneous administration of RTX aggravated the decline and resulted in profoundly delayed B-cell repopulation. We were able to reproduce these findings, as patients with preexisting HG continued to exhibit low IgG levels in our study. In our cohort, only six HG patients received RTX within 6 months of CYC exposure, and simultaneous treatment was rarely used; this may explain why CYC could not be identified as a predictor. Tariq et al. observed that nadir IgG levels occur during induction dosing and that IgG levels either increase or remain stable under maintenance dosing (24). Though not statistically significant, we observe a higher likelihood of HG after the RTX induction dose; and cases with severe HG at RTX start continued to exhibit low IgG levels later on.

The risk of developing severe infection in AAV is well established (29, 30), and an association between HG and severe infection in AAV was reported by several studies (11, 13, 31). We did not observe such an association, which we primarily attribute to the extensive use of PJP-prophylaxis with trimethoprim–sulfamethoxazole (TMP/SMX), as recommended in current and earlier guidelines. Odler et al. demonstrated in their study of severe infections in the RAVE trial that low-dose TMP/SMX significantly reduced the risk of severe infection (29). The composition of our cohort might be an additional explanation, as we describe a population-based AAV cohort representing the whole spectrum of AAV from systemic severe disease to mild–moderate localized disease, and other cohorts might represent a selection of more severe disease.

The use of IGRT or antibiotic prophylaxis other than TMP/SMX is unusual in our patients, though we lack exact data on the frequency, but as we meet all patients in clinical follow-up, we believe the frequency to be in the low single-digit range.

Our study has some limitations. HG was identified based on a single IgG measurement. In addition, all data were collected retrospectively and may have been incomplete, resulting in missed data. The same applies to the type and occurrence of severe infections, for which the number of events was also low. Furthermore, detailed data on IGRT were lacking.

As in other studies, many patients had received other immunosuppressive therapies prior to RTX treatment; therefore, HG cannot be attributed to RTX alone. Due to the retrospective nature of the study and changes in documentation systems over time, exact data regarding doses and timing of other immunosuppressive therapies could not be obtained in all cases, representing a further limitation. Most studies on this topic include patients previously exposed to other immunosuppressive agents. Together with evolving treatment recommendations for AAV, including reduced use of CYC, increased focus on glucocorticoid-sparing strategies, and emerging treatment options such as complement inhibition, this highlights the need for contemporary studies evaluating RTX with less confounding from prior immunosuppressive therapy.

It can be hypothesized that ongoing and upcoming treatment changes may decrease the occurrence of HG. Another limitation is the comparatively short follow-up time; HG can develop after many years, and we lack the ability to identify those cases.

The strength of our study is the population-based cohort incorporating the whole spectrum of AAV, paired with the long follow-up and maintenance of the cohort by the same researchers.

In summary, we find that HG commonly occurs in RTX-treated patients, with most patients exhibiting mild-to-moderate HG, with the IgG level at RTX initiation serving as the only independent factor predicting the occurrence of HG. It is more common in patients having received prior immunosuppressives before RTX. In our cohort, a higher risk for severe infection in patients developing HG could not be observed. Continuous monitoring of IgG levels in RTX-treated patients is warranted to identify subgroups of patients in need of IGRT, antibiotic prophylaxis, tighter follow-up, or modification of treatment.

## Data Availability

The original contributions presented in the study are included in the article/supplementary material, further inquiries can be directed to the corresponding author.
